# Hepatobiliary scintigraphy in vasculitis of the gallbladder as a manifestation of polyarteritis nodosa: a case report

**DOI:** 10.1186/1757-1626-2-9300

**Published:** 2009-12-10

**Authors:** Bjoern Kitzing, Sandra O'Toole, Adrian Waugh, Jane Clayton, Neil McGill, Kevin C Allman

**Affiliations:** 1 Department of PET and Nuclear Medicine, Royal Prince Alfred Hospital, Missenden Road, Sydney, New South Wales, Australia; 2 Department of Anatomical Pathology, Royal Prince Alfred Hospital, Missenden Road, Sydney, New South Wales, Australia; 3 Department of Rheumatology, Royal Prince Alfred Hospital, Missenden Road, Sydney, New South Wales, Australia

## Abstract

**Introduction:**

Polyarteritis nodosa can on rare occasions manifest itself as vasculitis of the gallbladder. Patients typically present with right upper quadrant pain and are initially worked up for cholecystitis. The definitive diagnosis is then usually based on surgical and histopathological findings.

**Case presentation:**

In this case a 23-year-old Caucasian female presented with a 3 week history of right upper quadrant pain and fevers.

**Conclusion:**

The clinical pathway and imaging findings of a rare case of gallbladder vasculitis as a manifestation of polyarteritis nodosa are demonstrated.

## Introduction

We present the clinical pathway of a rare case of gallbladder vasculitis secondary to polyarteritis nodosa. The clinical pathway demonstrates the importance of the clinical presentation and the use of medical imaging in confirming the diagnosis.

## Case presentation

A 23-year-old Caucasian female presented with a 3 week history of right upper quadrant pain, fevers, bilateral ankle pain and swelling with an accompanying rash. Her past medical history consisted of a previous hospital admission 3 months earlier due to an acute inflammatory polyarthritis associated with fever. During that admission the patient had developed a maculopapular rash over the trunk and limbs. Skin biopsies of the right thigh were taken which showed leukocytoclastic vasculitis. Symptoms improved after commencement of prednisone and the patient was discharged.

**Figure 1:**
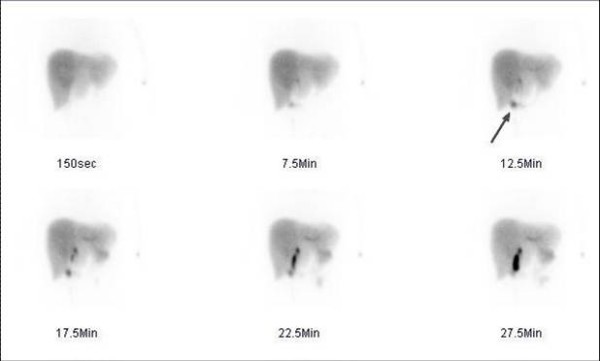
**Hepatobiliary scintigraphy of a 23-year-old white female with a three week history of right upper quadrant pain after injection of 220 MBq (5.9 mCi) Tc-99m disofenin**. Anterior dynamic views of hepatobiliary scintigraphy showing normal bile production with rapid appearance in the intrahepatic ducts and normal passage of bile into the bowel. A very small amount of activity entered the gallbladder early in the study (arrow).

**Figure 2:**
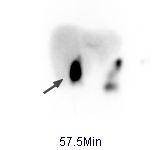
**Hepatobiliary scintigraphy of a 23-year-old white female with a three week history of right upper quadrant pain after injection of 220 MBq (5.9 mCi) Tc-99m disofenin**. Anterior delayed static view of hepatobiliary scintigraphy demonstrating progressive build up of activity in the gallbladder (arrow) after 57.5 minutes.

**Figure 3:**
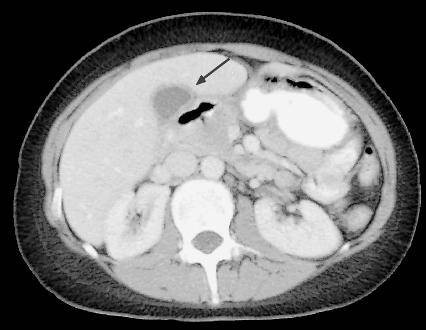
**Select axial image of abdominal computed tomography of a 23-year-old white female with a three week history of right upper quadrant pain showing a thickened gallbladder wall (arrow) with surrounding fluid**.

When the patient represented she had an elevated C-reactive protein 168 mg/L (normal: <12), erythrocyte sedimentation rate 76 mm/hr (0-12) and alkaline phosphatase 162 U/L (30-130). All her other blood results were normal. Rheumatoid Factor, ANCA and HLA B27 were negative as were blood cultures and viral serologies. Hepatobiliary scintigraphy was requested to investigate hepatobiliary function (Figures 1, 2). The study was performed with 220 MBq (5.9 mCi) Tc-99m disofenin. The vascular phase appeared normal. The parenchymal phase showed an enlarged left lobe of the liver. Bile production was normal with rapid appearance in the intrahepatic ducts and normal passage of bile into the bowel. However, the gallbladder filled slowly during the study. An outpatient computed tomography scan which had been performed prior to admission showed a thickened gallbladder wall with surrounding fluid but no evidence of stones or duct dilatation (Figure 3).

Based on these findings and the clinical picture a laparoscopic cholecystectomy was performed. The postoperative recovery was unremarkable and the patient was discharged two days later and subsequently had no further right upper quadrant pain. Clinical follow-up one month later showed that she was still free of abdominal pain but had developed symptoms consistent with mononeuritis multiplex. Polyarteritis nodosa was considered the most likely diagnosis clinically.

**Figure 4:**
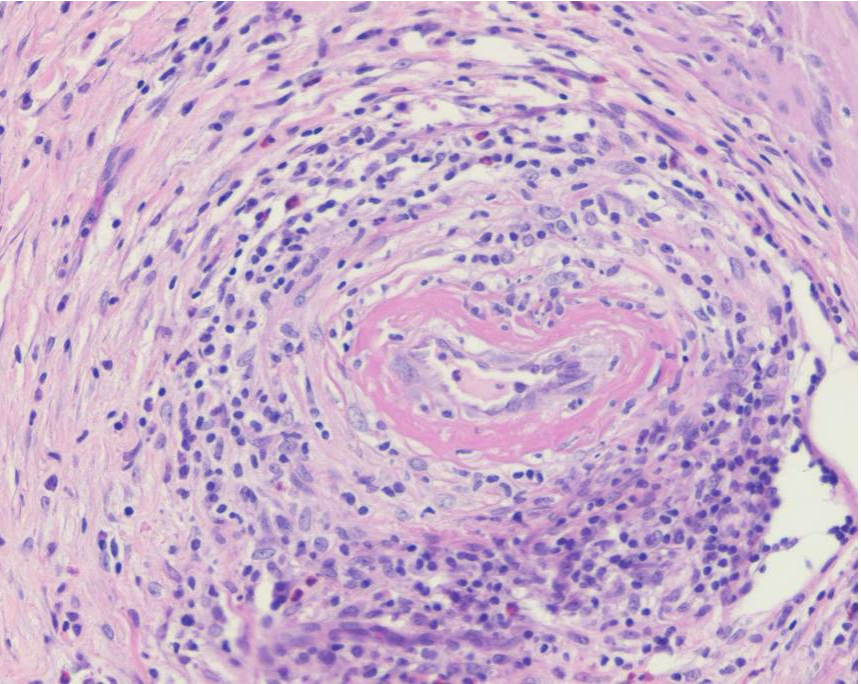
**Histopathology of the gallbladder of a 23-year-old white female with a three week history of right upper quadrant pain showing fibrinoid necrosis of a small artery, associated with lymphocytes and macrophages infiltrating the wall**. Occasional eosinophils are present. These findings are consistent with gallbladder vasculitis.

Macroscopic examination of the gallbladder showed no gross abnormality. However, microscopic examination revealed a striking vasculitis affecting small to medium sized vessels, predominantly arteries. There was fibrinoid necrosis of many vessels associated with an inflammatory cell infiltrate, composed predominantly of lymphocytes with very occasional eosinophils (Figure 4). Almost every vessel in the histological sections was affected and some showed thrombotic occlusion. The findings were consistent with polyarteritis nodosa.

## Conclusion

Polyarteritis nodosa (PAN) is an uncommon multisystem, necrotizing vasculitis of small- and medium-sized muscular arteries in which involvement of renal and visceral arteries is characteristic [1]. It was first described in its classic form by Kussmaul in 1866. The diagnosis of PAN is based on the demonstration of specific signs of vasculitis on biopsy material of involved organs as there are no diagnostic serologic tests. We present the hepatobiliary scintigraphy findings of a patient with cholecystitis which was a rare clinical manifestation of PAN.

Vasculitis is a clinicopathologic process characterized by inflammation of and damage to blood vessels. Polyarteritis nodosa (PAN) can cause specific complaints related to the vascular involvement within a particular organ system which may dominate the presenting clinical picture as well as the entire course of the illness. Involvement of the gastrointestinal tract has a 44% incidence in patients with classic PAN. Clinical manifestations include abdominal pain, nausea and vomiting, bleeding, bowel infarction and perforation, cholecystitis, as well as hepatic and pancreatic infarction. At autopsy 10% of patients with classic PAN have involvement of the gallbladder.

Gallbladder vasculitis of varying aetiology is rare but has been well described in the medical literature [2-13]. Abdominal pain, especially in the right upper quadrant is the most common presenting symptom. Liver function tests may be abnormal but as patients are usually thought to have cholecystitis secondary to cholelithiasis abdominal ultrasound imaging is performed sometimes followed by computed tomography [14]. Following laparoscopic or open cholecystectomy the diagnosis is then made based on the intraoperative and histological evidence.

In this reported case the hepatobiliary scintigraphy findings were consistent with slow filling of the gallbladder most likely due to oedematous swelling of the vasculitic tissue as was subsequently demonstrated by the histopathology. Appropriate knowledge and awareness of gallbladder vasculitis secondary to polyarteritis nodosa will help interpret non-specific yet clearly abnormal hepatobiliary scintigraphy findings.

## Abbreviations

ANCA: anti-neutrophil cytoplasmic antibody; HLA: human leukocyte antigen; PAN: polyarteritis nodosa; IDA: iminodiacetic acid.

## Consent

Written informed consent was obtained from the patient for publication of this case report and all accompanying images. A copy of the written consent is available for review by the Editor-in-Chief of this journal.

## Competing interests

The authors declare that they have no competing interests.

## Authors' contributions

BK, SOT and AW made substantial contributions to conception and design and drafted the manuscript. JC, NM and KCA revised it critically for important intellectual content and gave final approval of the version to be published.
